# Systematic Review and Meta‐Analysis: Taurine and Its Association With Colorectal Carcinoma

**DOI:** 10.1002/cam4.70424

**Published:** 2024-12-04

**Authors:** Akshat Sinha, Liam Griffith, Animesh Acharjee

**Affiliations:** ^1^ Institute of Cancer and Genomic Sciences University of Birmingham Birmingham UK; ^2^ MRC Health Data Research UK (HDR UK) Birmingham UK; ^3^ Institute of Translational Medicine University Hospitals Birmingham NHS, Foundation Trust Birmingham UK; ^4^ Centre for Health Data Research University of Birmingham Birmingham UK

**Keywords:** biomarker, colon cancer, metabolomics, NMR, taurine

## Abstract

**Background:**

Colorectal cancer (CRC) is one of the most common cancers. Various options are available for treatment, but prognosis is still poor in the more advanced stages. Current screening methods are not as accurate for distinguishing between benign and malignant growths, resulting in unnecessary invasive procedures. Recently a focus has been placed on identifying metabolites. Of these, taurine has frequently been detected, and this particular compound has a multifactorial role in human physiology.

**Methods:**

We conducted a systematic review of studies up till November 2023. Searches were done in three databases‐ MEDLINE, CINAHL‐Ebsco, and PubMed. Three independent reviewers filter titles, abstracts, and full‐texts according to selection criteria. Ten studies (samples = 1714) were identified showing a differential level of taurine in CRC patient samples. Quality assessment accounted for the risk of bias of each study using the ‘robvis’ tool. Where meaningful comparisons could be made, meta‐analyses were carried out using the ‘R' program for precalculated effect sizes with ‘metagen’ in R. The ‘meta’ package was utilised for creation of forest plots.

**Findings:**

Taurine was shown to significantly increase odds of CRC. It was also significantly associated with being a discriminator for CRC as a diagnostic metabolite. This was maintained at various stages of CRC. Taurine had increased expression in CRC patients, especially when the matrix utilised was blood. Nevertheless, there was significant heterogeneity for some outcomes.

**Interpretation:**

In conclusion, these findings highlight the potential of using taurine as well as other bile acid metabolites (lithocholic and ursodeoxycholic acid) to diagnose CRC and illustrate the link with microbiome interactions. Overall increased taurine concentration are associated with significantly increased odds for CRC. There was mostly an increase in relative expression of taurine in CRC samples, excluding results from Wang et al.

## 1 Introduction

Colorectal carcinoma (CRC) is one of the most common cancers to affect both men and women. Recently the incidence of cases has been increasing worldwide due to a variety of factors [1, 2]. Amongst both sexes it is the third leading cause of cancer death [3]. In the UK over 40,000 people are diagnosed with CRC each year, and over a million globally [4, 5]. This most commonly occurs in the elderly population with screening taking place using the faecal immunochemical test (FIT) for those between 54 and 74 years in the UK. This is gradually expanding to include those in the 50–53 year bracket as well. Unfortunately, the majority of the cases are diagnosed at an advanced stage, at which survival rates are fairly low despite medical and surgical intervention [6].

Risk factors that can cause the development of CRC include: obesity, smoking, a diet high in red/ processed meat, and a diet low in vegetables and fruit. Traditionally these aforementioned risk factors were present more frequently in highly developed countries [2, 7]. However, with the rise of globalisation we have observed a much more even spread of new cases [7, 8].

**FIGURE 1:**
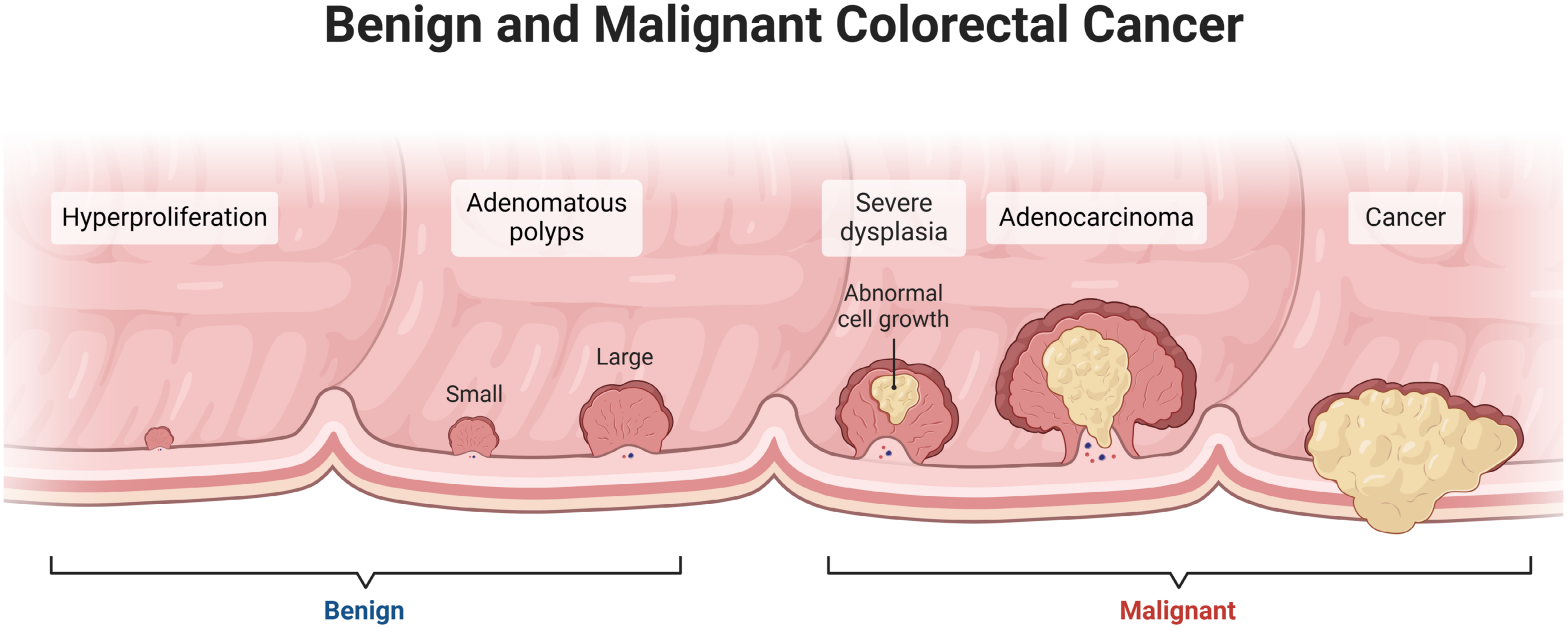
Steps in development of colorectal cancer. First benign growths know as adenomas start to form before the cells eventually become dysplastic and change into adenocarcinoma. At first this is localised, but over time can spread and invade through the layers of the bowel wall [53].

Although surveillance techniques and screening programmes exist, these existing screening programmes pose a major burden on an already overwhelmed healthcare system. More accurate early detection tools with high specificity are required to prevent unnecessary downstream invasive procedures such as colonoscopy and biopsy [9]. For most, the results are mostly polyps rather than malignancies [10] (Figure 1). As shown in Figure 1, these lesions progress over time. Stage 0 is when the cancer is situated on top of the innermost layer‐ the mucosa. Stage I occurs after invasion into this mucosa. Subsequently stages II and III reflect extension into the muscular and outer layer extensively. Lymph node involvement begins at stage III. The final stage IV is the resultant distant metastasis after spreading beyond the bowel tissue. There is a need for more targeted methods to identify those who are at higher risk, in order to stratify individuals for further testing [11].

Besides specificity, sensitivity is an important characteristic of early detection tools. The tool should identify individuals with precancerous lesions that may be overlooked with current monitoring processes [12]. Moreover, biomarkers that can indicate stage can have predictive value when it comes to prognostication [13, 14]. Improved staging can make more effective use of the service and resources available. Separating the population into clusters can lead to personalised medicine where care is based on individual characteristics.

Molecular data is very important to understand mechanisms in colon cancer as it is multifactorial in nature. From a genetic standpoint a plethora of pathways occurring during the cell cycle can become affected [15]. Many genes, transcription factors, lipids and metabolites are involved in this process. Examples of this are seen in many inherited familial conditions such as familial adenomatous polyposis (FAP), lynch, and Gardner syndrome. One of the key aspects is the interaction between amino acids and other microbes/genes. Multi‐omics has enabled us to explore this further to pinpoint differential genes and metabolites [16, 17]. Likewise, examination of the microbiome has elucidated how imbalances here can also promote the growth of CRC [18, 19].

## 2 Research in Context

### 2.1 Evidence Before This Study

Adenocarcinoma is the most common form of colorectal cancer. Many biomarkers have been identified that are associated with CRC such as carcinoembryonic antigen (CEA). In addition to this, in recent years multiple metabolites have been identified using metabolomics that have positive associations with CRC [20, 21]. Of these metabolites branched‐chain amino acids (BCAAs) e.g. isoleucine, leucine, valine have been investigated utilising faecal and urinary samples. As well as cancer, certain types of inflammatory bowel diseases such as ulcerative colitis can increase the risk of CRC [22, 23]. Furthermore, the immuno‐inflammatory response has been linked to the initiation and progression of malignant processes [24, 25].

### 2.2 Added Value of This Study

A novel function of taurine has been reported in relation to CRC. Taurine has been shown to reduce the immune response as well as dampening the production of pro‐inflammatory cytokines [26]. Much of the function of taurine is classed into bile acid synthesis pathways. Its anti‐tumorigenic nature can inhibit cell proliferation and induce apoptosis of cancer cells [27, 28]. Overall, though there are still queries remaining about the exact mechanisms by which this occurs. As a result, it has become a topic of intense interest as to whether it has any effect on CRC similar to other amino acids. A few large trials have been conducted evaluating amino acid profiles linked to CRC, such as the EPIC cohort from the UK Biobank [29].

### 2.3 Implications of Available Evidence

In this study, we have performed a comprehensive systematic review looking at taurine association with colon cancer. Our objectives are to determine the effect of the amino sulfonic acid taurine in colorectal carcinoma samples versus healthy controls. Taurine is not an essential dietary nutrient, however its role is pivotal for retinal health, bile salt formation as well as reducing heart failure [30]. Using results from multiple studies will allow us to conduct statistical analyses. Doing so will help in exploring the potential correlations between taurine and malignant neoplasm.

Then we will observe if taurine does increase the development of CRC. Producing fold change in taurine expression between the two groups will enable us to carry out a meta‐analysis. This will be the one of the first and most recent study to perform a meta‐analysis in relation to taurine levels. Currently based on various research on the function of taurine, our hypothesis is that taurine levels are lower in healthy controls compared to colorectal cancer samples. Lower taurine levels are protective.

## 3 Methods and Analysis

### 3.1 Search Strategy and Criteria for Inclusion

A systematic review and meta‐analysis were conducted. We searched the following databases: PubMed, MEDLINE, and CINAHL‐Ebsco. Relevant studies from inception to 06/11/2023 were eligible for selection. Citations were analysed to identify further studies for inclusion. Other sources such as Google Scholar were manually searched to increase the number of available studies for screening. References on included studies were checked to identify other potential articles for inclusion. No limits were placed upon these searches, and the results are depicted as per the ‘preferred reporting items for systematic reviews and meta‐analyses (PRISMA) guidance [31].

Those accepted post full‐text review were those carried out using human samples, which assessed the diagnostic capabilities of the amino sulfonic acid taurine. Selection and assessment involved two reviewers working separately (AS and LG). Registration took place on Prospero (International Prospective Register of Systematic Reviews) prior to data extraction, ID: CRD42024507334 [crd.york.ac.uk/prospero/display_record.php?RecordID=507334].

**FIGURE 2:**
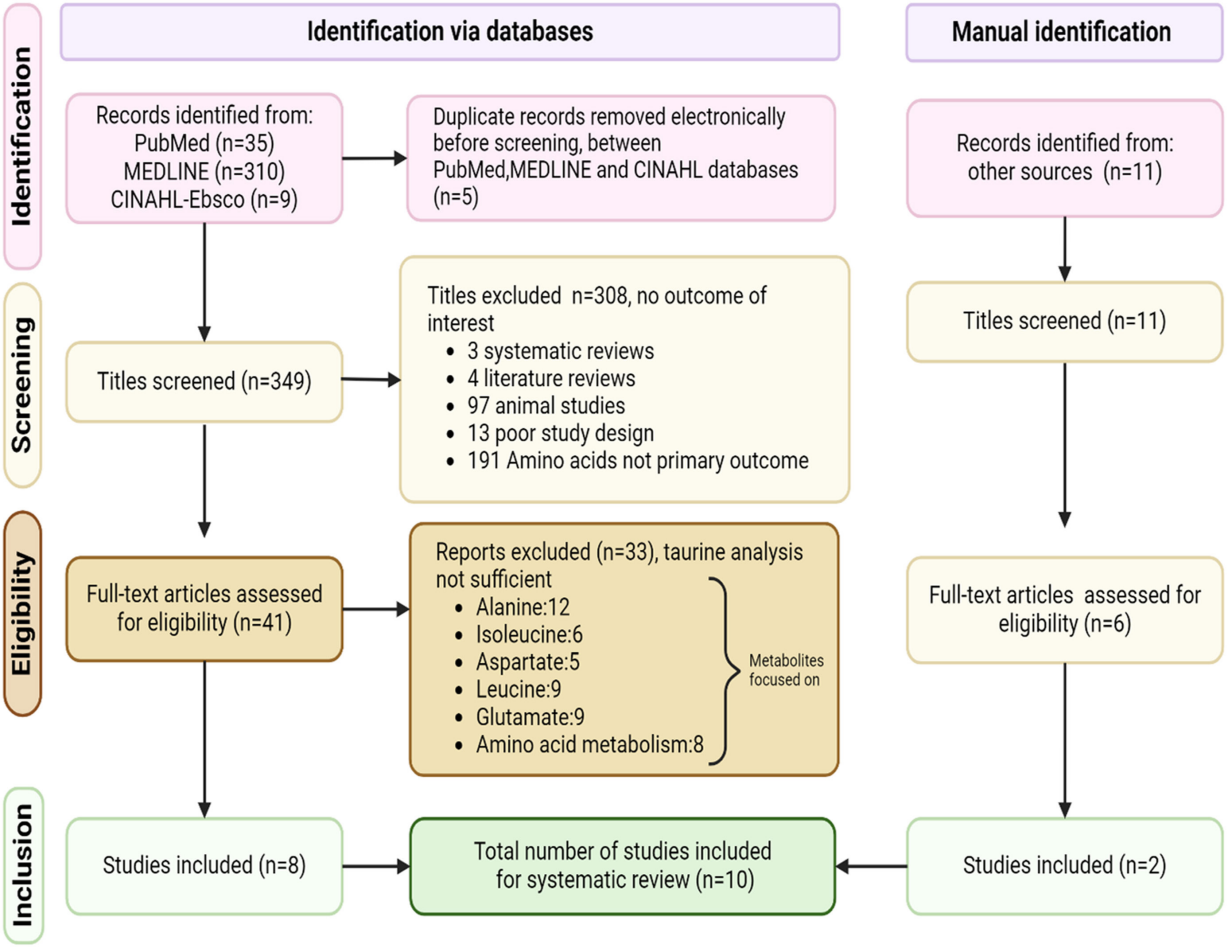
PRISMA flowchart depicting screening and eventual selection of studies. All studies must have had taurine as a differentially expressed metabolite, for analysis purposes. Animal studies or isolated cell lines were not considered.

Age or gender restrictions did not apply to our study characteristics for inclusion. All study types were considered such as randomised controlled trials (RCT), cohort, and case–control studies. Additionally, a comparator group of healthy controls or adenomatous tissue (benign adenomas) was essential to be included in our meta‐analysis. Regarding techniques, metabolomics studies utilising GC/LC–MS (gas/liquid chromatography–mass spectrometry) and nuclear magnetic resonance (NMR) were suitable for inclusion. Some of the reasons for exclusion were the use of murine/animal samples and those that did not have sufficient information regarding taurine for thorough analysis. These related to other BCAAs from the period 2013 to 2017 and were excluded (Figure 2).

Initial screening took place by title and abstract. Decisions were made independently by two separate reviewers (AS and LG). Then any conflicts were settled through a group discussion with a third reviewer (AA) also present. A similar process occurred for full‐text review prior to data extraction.

#### 3.1.1 Search Strategy

1. colon cancer.mp. or exp. Colonic neoplasms/
2. colorectal cancer.mp. or exp. Colorectal neoplasms/
3. 1 or 2
4. exp. Amino Acids/or exp. Metabolomics/or amino acid metabolism.mp. or exp. Metabolome/
5. exp. Isoleucine/or isoleucine.mp.
6. taurine.mp. or exp. Taurine/
7. leucine.mp. or exp. Leucine/
8. alanine.mp. or exp. Alanine/
9. 5 or 6 or 7 or 8
10. 3 and 4 and 9

#### 3.1.2 Inclusion Criteria

- Taurine must be investigated either as biomarker, or as concentration in samples.
- Human tissue **OR** Serum/Faecal/Urine samples.
- Must include healthy control **AND** cancerous samples, with/without adenoma samples.
- Cancerous samples from anywhere in colon e.g. ^**1**^caecum, ^**2**^rectum, ^**3**^sigmoid region.
- Focus on biomarker identification as opposed to mechanism.
- Analysed by NMR/MS.

### 3.2 Methodological Quality Assessment

Risk of bias in the included studies was assessed using the Quality Assessment of Diagnostic Accuracy Studies‐2 (QUADAS‐2) assessment tool [32]. This was done in duplicate by two reviewers (AS & LG). LG acted as an adjudicator where there was a difference of opinion. The components of QUADAS‐2 examined were the index test, reference standard, patient selection, flow and timing. Index tests should have been interpreted blind to results of the reference standard. Regarding the reference standard this test is assumed to have a 100% accuracy in detecting the target condition. Patient selection should ideally be random. If not a consecutive sample should be obtained with limited exclusion, unless necessary. Flow and timing refers to the proportion of patients receiving the index test and reference standard. Also the delay between the two tests, and if this could potentially affect the results.

Three categories were used to assess each of these domains; either showing ‘high,’ ‘low’ or unclear risk of bias. A label of unclear risk was reserved for studies providing insufficient information about a certain domain. If two aspects involving a domain were seen as being absent, then the overall risk for that domain was also deemed as being high. The ‘robvis’ tool in ‘R' was used to create a visual representation of the methodological quality.

Funnel plots were not produced to evaluate publication bias or small‐study bias, as we were not able to pool more than ten studies for the outcomes investigated.

### 3.3 Data Extraction

Analysis took place initially on an Excel spreadsheet before moving onto appropriate meta‐analysis software. Data was entered into a pre‐specified template in the shape of a digital extraction form. General information was also collated regarding date of publication, article type, study type and author information. Two reviewers were involved in the process (AA & AS) with cross‐checking by another author (LG) to verify results.

Information on the outcome of CRC was collected according to taurine exposure. The collection process also involved interpreting sensitivity and specificity values from receiver operating characteristics (ROC) curves. This allowed us to extract area under the curve (AUC) values where provided, to assess the quality of results. As well as this the raw concentration of taurine in malignant and healthy tissues were compared. Doing so meant relative risk was calculated and *p‐*values were produced to assess significance.

Where there was insufficient information, we contacted study investigators to obtain missing data or verify existing materials. If this was not possible and quantitative values were not available, these studies were not included in the meta‐analysis aspect of this review.

### 3.4 Data Synthesis and Statistical Analyses

Statistical analysis was carried out in R (version 4.3.3) [33] using the ‘meta’ package [34] to create the forest plots comprising the meta‐analysis. Stata/SE (version 18.0) was also used to create forest plots with precalculated effect sizes. The corresponding tool in ‘R' was the ‘metagen’ tool part of the ‘meta’ package. This was done for studies reporting odds ratio and fold change in taurine. 95% confidence intervals (95% CI) were also calculated for each study. Effect sizes were pooled using the inverse variance method. Due to the anticipated heterogeneity across the studies a random‐effects model was used. Weights attributed for each study in the meta‐analysis were derived from this model.

The closer to 1 the AUC was, the better taurine was as a diagnostic marker of CRC. Odds ratio of more than 1 suggested the risk of CRC was increased with elevated taurine, and vice versa for less than 1‐ those with CRC had greater odds with higher levels of taurine. OR represents the relative change in CRC risk when there is a one standard deviation (SD) change in metabolite intensity.

Fold change indicated the relative increase or decrease in the expression of taurine comparing samples from cancer patients to healthy controls. Heterogeneity was assessed using Tau^2^ and *I* ^2^. *I* ^2^ is the percentage variability in effect sizes due to methodological differences. While Tau^2^ is an absolute measure of heterogeneity of the true effect sizes between studies, *I* ^2^ ranges from 0% to 100%, and the heterogeneity was based on this value. Results with a value of 0%–25% had low heterogeneity, 25%–50% had moderate heterogeneity and > 50% was considered to be substantial heterogeneity. Paule‐Mandel (PM) was the technique used to calculate Tau^2^ using the random‐effects model. A *p‐*value of < 0.05 was considered to be a significant result. This was based on a two‐tailed distribution. When confidence intervals were not provided, standard error was estimation using *p‐*values and their corresponding *z‐*values. Using the effect size, upper and lower bounds for 95% confidence intervals were calculated. All data is presented in absolute form rather than on a logarithmic scale, to aid interpretation and add more meaningful results to findings.

### 3.5 Data Availability Statement

All data produced in this review is available in the original trials and articles. Extraction tables and meta‐analysis data is free on request. There are no declaration of interests to request. Results are not to be used for commercial purposes, and no funding was received in the production of this review.

## 4 Results

### 4.1 Study Selection

Our searches yielded 354 articles from three databases. After removal of five duplicate results, 349 articles were screened in the title and abstract stage. Of these, 41 records were retrieved for full‐text review. Eight of these met the criteria required for inclusion in our review. Those excluded did not have sufficient information regarding taurine. They focused on other substances detailed in the flowchart above. As a result, they were excluded. Eleven studies were identified from other sources, of which two were included in this review. This left ten studies for quantitative and qualitative analysis (Figure 2). The ten studies comprised of a mixture of prospective cohort and case–control studies.

The ten studies identified were published between 2012 and 2021 (Tables 1 and 2). They used a mixture of matrices, and a variable number of healthy and neoplastic samples [35, 36, 37, 38, 39, 40, 41, 42, 43]. Studies were both retrospective and prospective sampling known cases of colon cancer.

### 4.2 Methodological Quality Appraisal (Risk of Bias)

**FIGURE 3:**
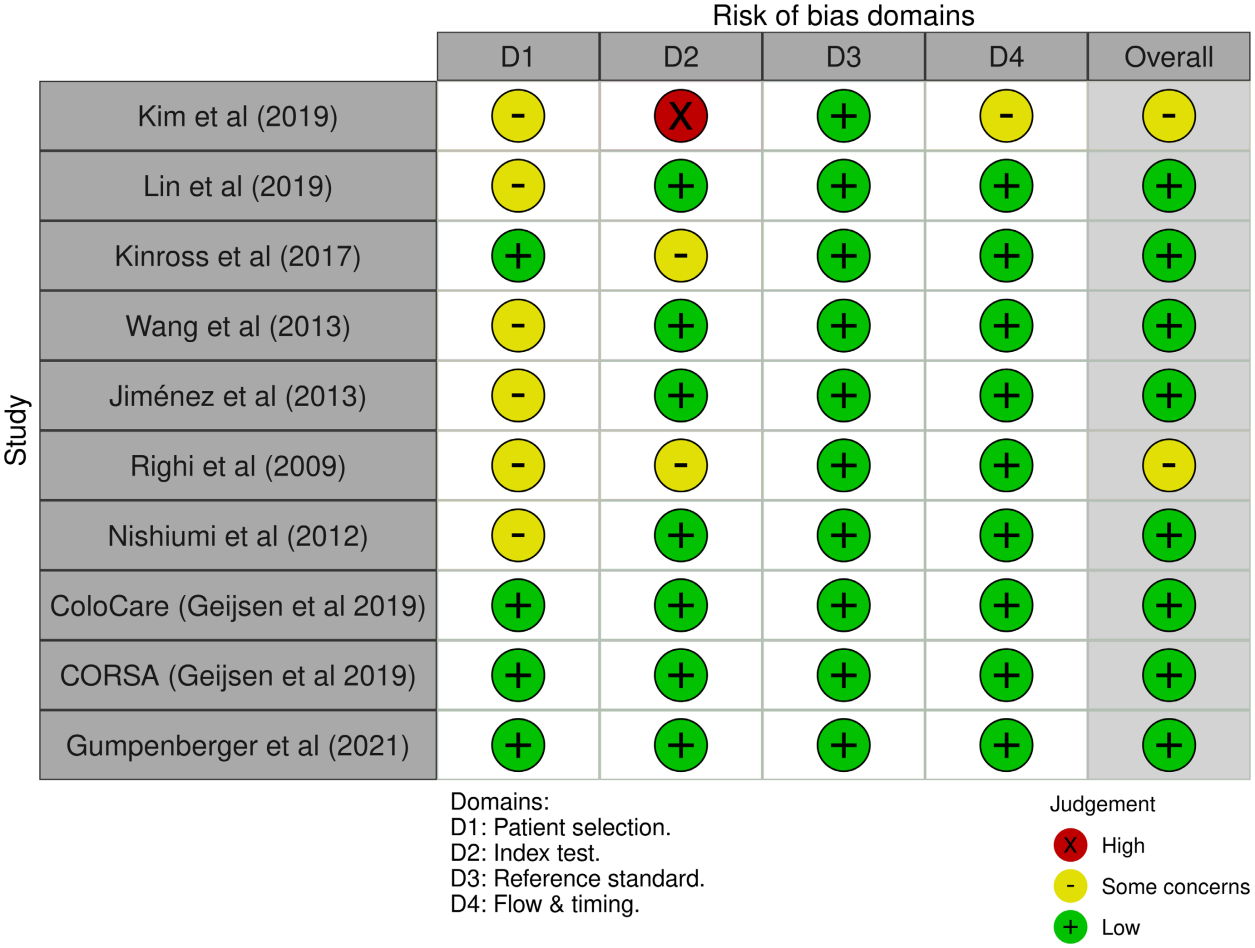
Risk of bias plot: QUADAS‐2 Domains (10 studies assessed across four domains for bias, red = high risk, yellow = some concerns, and green = low risk).

Risk of bias assessment for all studies were conducted according to QUADAS‐2 domains for diagnostic test accuracy (DTA) studies (Figure 3). The domains were patient selection, index test, reference standard and flow/timing. In general, there was an overall low risk of bias. Six studies had some concern for domain one, as they were retrospective case–control studies.. Kim et al. had a high risk of bias for the index test because it was interpreted with knowledge of the outcome. On the whole, the reference standard in addition to flow and timing of experiments were conducted in a rigourous fashion.

### 4.3 Characteristics of Included Studies

**TABLE 1 |:** Baseline characteristics of included trials.

Study	Design	Group	Age (years)	Male gender (%)	BMI (kg/m^2^)
Kim et al. [35]	Case–Control (South Korea)	CRN = 92	60 (32–85)	62 (67)	23.6 (18.3–33.4)
		HC = 156	52 (22–76)	76 (49)	23.0 (16.9–34.6)
Lin et al. [36]	Case–Control (China)	CRC = 50	ND	ND	ND
		HC = 50			
Kinross et al. [37]	Prospective Cohort (UK)	CRC = 18	76 (55–85)	10 (56)	26.6 (21.0–39.0)
Wang et al. [38]	Case–Control (China)	CRC = 127	55 (28–86)	69 (54)	ND
		HC = 43	56 (35–85)	16 (37)	ND
Jiminez et al. (2013) [39]	Prospective Cohort (UK)	CRC = 26	72 (26–87)	10 (38)	ND
Righi et al. [40]	Case–Control (Italy)	CRC = 14	61 (52–70)	7 (50)	ND
		HC = 9	63 (54–72)	3 (33)	ND
Nishiumi et al. [41]	Case–Control (Japan)	CRC = 60	70 (36–88)	39 (65)	21.7 (20.2–24.0)
		HC = 60	68 (39–88)	39 (65)	21.9 (20.3–23.6)
ColoCare (Geijsen 2019) [42]	Prospective Cohort (Germany)	CRC = 180	66 (58–73)	114 (63)	26.4 (24.1–29.2)
		HC = 153	51 (42–63)	59 (39)	23.5 (22.0–26.7)
CORSA (Geijsen 2019) [42]	Prospective Cohort (Austria)	CRC = 88	70 (60–76)	60 (68)	26.1 (23.8–29.4)
		HC = 200	64 (57–74)	130 (65)	27.0 (24.9–30.1)
Gumpenberger et al. [43]	Prospective Cohort (Austria)	CRC = 88	70 (60–76)	60 (68)	26.1 (23.8–29.4)
		CRN^a^ = 200	65 (56–73)	132 (66)	27.3 (24.3–30.0)
		CRN^b^ = 200	66 (55–73)	132 (66)	27.2 (24.6–30.9)

Abbreviations: CRC, colorectal cancer; CRN, colorectal neoplasia; HC, healthy control.

^a^ High risk adenoma.

^b^ Low risk adenoma, BMI = body mass index, ND = not disclosed.

All data is displayed as the median and interquartile range unless stated otherwise.

**TABLE 2 |:** Summary of findings table.

	AUC	*p*‐value	Fold changes	Matrix
Kim et al. [35]	0.82 (0.77–0.87)	< 0.0001	Increase	Urine
Lin et al. [36]	0.90 (0.78–0.96)	< 0.001	1.65↑ CRC	Tissue
Kinross et al. [37]	ND	< 0.0001	Increase	Tissue
Wang et al. [38]	ND	0.001	Decrease −2.1 CRC VIP 4.25	Tissue
Jiminez et al. (2013) [39]	ND	7 × 10^−9^ 0.03	↑1.8× CRC ↑1.3× adjacent tissue	Tissue
Righi et al. [40]	ND	ND	ND	Tissue
Nishiumi et al. [41]	ND	0.0001 0.0001 0.11	3.43–0 to 4 4.63–0 to 2 1.99–3 to 4	Blood
	AUC	*p*‐value	Odds ratio	Matrix
ColoCare (Geijsen 2019) [42]	ND	< 0.01	1.44 (1.13–1.87)	Blood
CORSA (Geijsen 2019) [42]	ND	< 0.0001	1.95 (1.53–2.31)	Blood
Gumpenberger et al. [43]	ND	< 0.0001	16.17 (7.81–35.24)	Tissue

Abbreviations: AUC, area under curve; CRC, colorectal cancer; CRN, colorectal neoplasia; HC, healthy control; ND, not disclosed; VIP, variable importance in projection.

### 4.4 Diagnostic Aspects of the Metabolites Using ROC Curves (AUC) (Table 3)

**TABLE 3 |:** AUC diagnostic accuracy of taurine as biomarker for CRC.

	AUC	Matrix	Diagnostic target
Kim et al. [35]	0.823 (0.768–0.870)	Urine	Precancerous adenoma/Stage 0
Lin et al. [36]	0.896 (0.777–0.964)	Tissue	CRC Stage I–IV

Abbreviations: AUC, area under curve; CRC, colorectal cancer.

Two studies investigated AUC and ROC curves to assess the suitability of using taurine levels to distinguish between CRC and benign samples. Kim et al. found that the relative amount of taurine in patients with colorectal neoplasia (CRN) was greater than in healthy controls using urine NMR metabolomics [35]. Taurine was not the only metabolite increased, with examples of alanine and valine having an increased concentration in CRN as well. This change was observed across all stages of CRC, ranging from adenoma to advanced cancer (*p* = 2.86 × 10^−4^). Taurine had a high AUC score of 0.823 (95% CI: 0.768–0.870). Multiple ROC curve analysis demonstrated the highest ranking for taurine as a discriminator for CRC, amongst the differentially expressed metabolites. Lin et al. also reported a high AUC value of 0.896 (0.777–0.964) for taurine. In contrast this study utilised CRC tissue samples [36]. Again, the increased levels of taurine distinguished CRC tissue from adjacent and distant non‐cancerous samples (*p* < 0.001).

Pooling together the two studies that had calculated an AUC value led to an average AUC of 0.849. A random‐effects model was used to account for the heterogeneity. There was moderate heterogeneity in the results seen (*I* ^2^ = 44.6%) but this was not significant (*p* = 0.179). Overall, the high value seen suggests that taurine at worst has an AUC value of 0.777 based on the lower limit of the confidence interval. The differences in AUC may be caused by the influence of matrices used on the metabolic profile (urine in Kim et al., tissue in Lin et al.).

### 4.5 Fold Change (FC) in Taurine Expression

**FIGURE 4:**
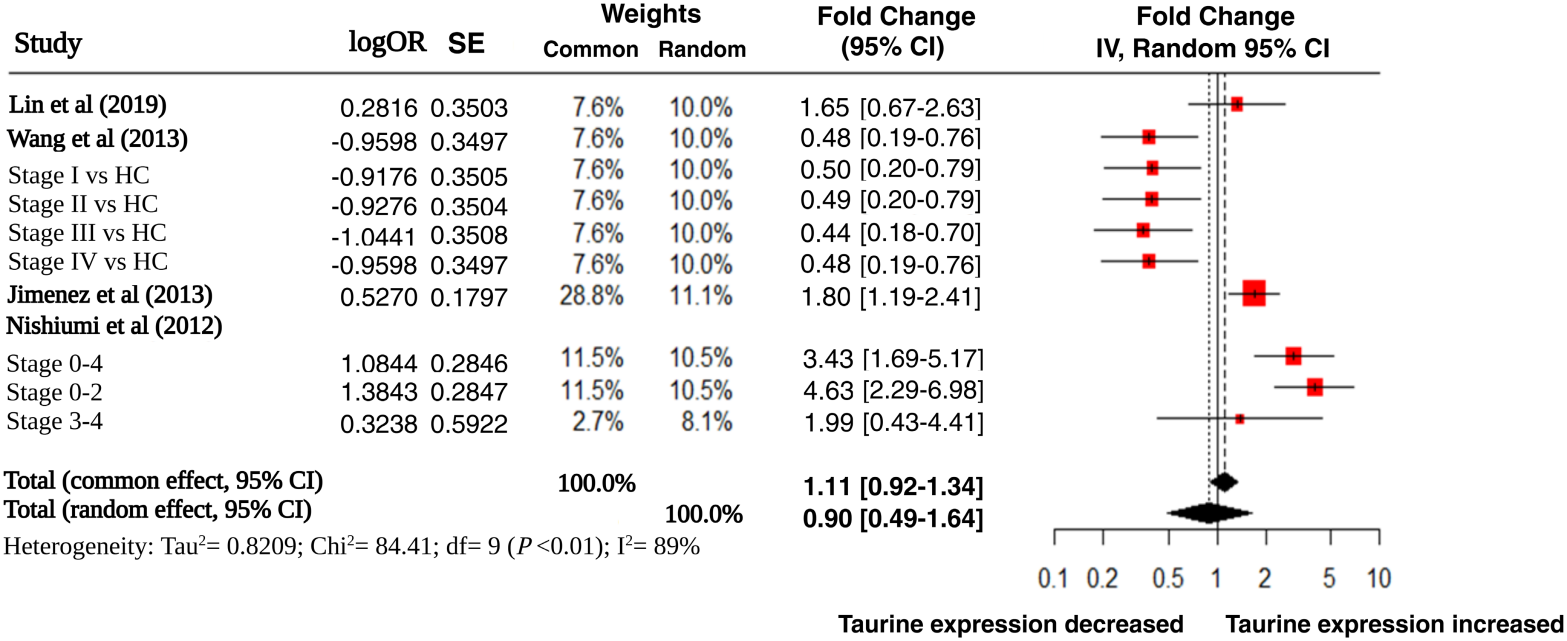
Forest plot‐ fold change of taurine in healthy control Vs colorectal cancer samples (logOR = logarithm of odds ratio, SE = standard error, common and random‐effects models used to account for methodological differences with corresponding weights, IV = inverse variance).

Four studies also looked at the fold change in taurine expression in CRC patients compared to healthy controls. Furthermore Wang et al., and Nishiumi et al. also stratified according to the stage of CRC [38, 41]. All the studies apart from Wang et al. demonstrated an increased fold change in taurine, in patients with CRC. Wang et al. found a decrease in taurine by 2.1 times in CRC samples. The variance importance in projection (VIP) was 4.25 suggesting a strong difference between the two cohorts. This difference was maintained across the different stages of CRC as well, in this particular study (Figure 4).

The overall pooled value indicated increased taurine when applying the common effects model. However with the random‐effects model this was not significant (fold Change 0.9). As well as this the common effects model also crossed the threshold of insignificance.

There was a substantial level of heterogeneity (*I* ^2^ = 89%, *p <* 0.01). Perhaps this was expected as results from Wang et al. will have increased the level of statistical heterogeneity due to its contrasting results compared to all the other studies. For the common effects model, the effect size was FC 1.11 (95% CI 0.92–1.34). and the *p*‐value was 0.2978. With the random‐effects model, the *p‐*value was 0.72, suggesting no relative change in taurine expression between CRC and healthy control samples (Figure 4). After exclusion of the results from Wang et al., heterogeneity was reduced but still moderate (*I* ^2^ = 60.7%, *p* = 0.037). With all other studies showing increased expression of taurine, the pooled FC was 2.12 (*p* < 0.0001) and 2.16 (*p* = 0.0004) using the common and random‐effects model, respectively.

### 4.6 Odds Ratio (OR)

Odds ratio was investigated in three studies. All of them made use of the ColoCare and CORSA trials. The study by Geijsen et al. focused on the discovery and replication stage of CRC [42]. Here, taurine has a positive association with CRC in the discovery stage (ColoCare). A standardised odds ratio of 1.44 (95% CI 1.13–1.87) was identified. The relative intensity values for taurine were 12.53 ± 0.28 and 12.41 ± 0.24 in CRC and control patients respectively (*p* = 7.2 × 10^−3^). Similarly in the replication stage which focused on results from the CORSA cohort, taurine was also significantly associated with increasing the odds of CRC. In this case OR was higher (1.95; 95% CI 1.53–2.31, *p* = 4.2 × 10^−6^) with a more significant increase in the development of CRC. For the replication cohort relative intensity values were 12.96 ± 0.49 in CRC samples, and 12.66 ± 0.34 in healthy controls. The *p*‐values were corrected accounting for the false discovery rate (FDR). As a result, technically these are referred to as *q*‐values.

**FIGURE 5:**
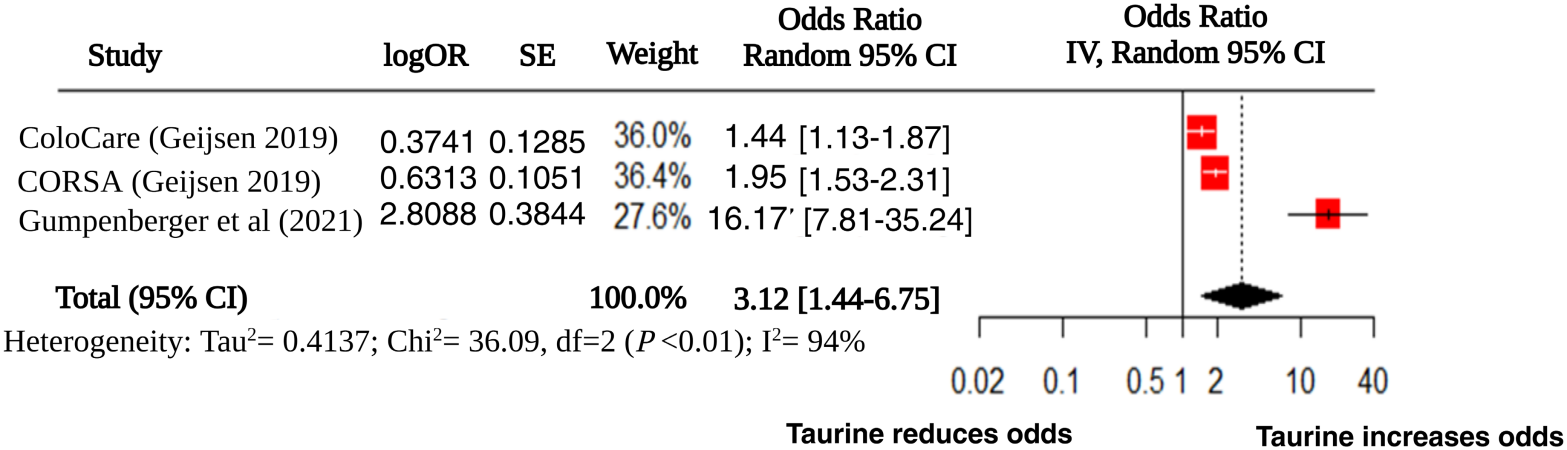
Forest plot‐ standardised odds ratio for development of colorectal cancer (logOR = logarithm of odds ratio, SE = standard error, common and random‐effects models used to account for methodological differences with corresponding weights, IV = inverse variance).

Subsequently, Gumpenberger et al. carried out an additional analysis on the CORSA cohort [43]. Rather than looking at CRC versus control, they used: CRC, high risk adenomas and low risk adenomas. Likewise, as for CRC versus control; taurine was associated with increased risk of CRC compared to both low and high risk adenomas. The OR was much larger (16.17) with a highly significant *q*‐value (6 × 10^−13^). When pooling the three studies the overall OR was 3.12 (95% CI 1.44–6.75; *p* = 0.0038) (Figure 5). There was a high amount of heterogeneity (*I* ^2^ = 94%, *p <* 0.01), probably because of the skewed results from the Gumpenberger et al. study.

## 5 Discussion

To our knowledge this is the first systematic review and meta‐analysis looking at the relation of taurine and CRC. Taurine is an antioxidant that can be produced in high concentrations in environments with increased oxidative stress i.e. tumour microenvironment [44]. Taurine has a role in microbiome mediated interactions, while the composition of the gut microbiota is also linked to development of CRC [45]. It has also been hypothesised that taurine is used as a fuel source for cell proliferation via the tricarboxylic acid (TCA) cycle [46]. Multiple factors can contribute to imbalances in the microbiome. Dysbiosis can occur due to geography, diet, age, disease, exposure to drugs, and level of physical activity. Recently using precision medicine, analysing the microbiome has led to it being used as a diagnostic tool. Communication happens across the gut‐brain axis with numerous physiological systems becoming affected when there is a negative change in the balance of microbial diversity. Immunity is an important role maintained by the control of this axis [47, 48].

In total, ten studies were included for both quantitative and qualitative analysis. Baseline characteristics were not available for the study conducted by Lin et al. [36]. The studies ranged from prospective cohort studies to case–control studies. There were 1714 samples from patients. Matrices examined included: urine, tissue, and blood.

Regarding the AUC, taurine displayed a high sensitivity and specificity to discriminate between healthy controls and CRC and various stages of cancer. The amount of taurine was also increased in cancerous tissue. Critically, this change was observed both in urine and tissue samples. For the tissue samples, distant benign samples were collected minimising the chances of contamination.

There was more heterogeneity in results for fold change, however. Wang et al. was the only study that showed decreased taurine expression at all stages of CRC, and the confidence intervals did not cross threshold [38]. The other studies showed increased expression however some of them were not entirely significant. Consequently, the overall pooled estimate was also not significant in favour of increased expression. One aspect to take into account is that Nishiumi et al. demonstrated the largest increases in fold change and used blood samples in contrast to the others included in this meta‐analysis that collected tissue samples [41]. Looking at this isolated result could show that there may be different levels of taurine in blood compared to the tissue in CRC patients. Also, it can depend on where exactly the tissue samples were taken from and how close the healthy tissue was to the CRC tissue.

Subsequently, the three prospective cohort studies calculated standardised odds ratios. This time, taurine increased the odds of developing CRC significantly. Regardless of matrices utilised, either blood or tissue, the odds were significantly increased. Interestingly, again the Gumpenberger et al. study which used blood samples, showed much increased odds similar to the pattern observed for fold change [43]. This was despite the fact that in this study CRC was being compared to adenomas, not even healthy controls. Once more this could suggest that there is a difference depending on the matrix used for analysis.

Moreover, the potential for taurine is not limited to detection of CRC. It can also apply to other cancers in the gastrointestinal tract. One such example is that of pancreatic cancer, where short chain fatty acids (SCFAs) were dysregulated in cases of pancreatic adenocarcinoma [49]. The bacterial diversity in a number of pancreatic fluids including bile was significantly lower in another study [50]. This thereby highlights the utility of modifying the microbiome to improve the composition of the gut microbiota and improve health. Doing so encourages personalised treatment approaches, via a variety of therapeutic options. Similarly there is evidence to support taurine's use in non‐cancerous conditions such as inflammatory bowel disease (IBD) [27]. Separate to this is the general anti‐inflammatory and antioxidant effects linking taurine with reduced cardiovascular events, as well as ageing [51].

All these applications can have positive implications for chronic inflammation driven diseases. Translational medicine has provided a route where machine learning can rapidly identify these avenues for further research [52, 53]. Since we are moving more towards precision medicine, vast amounts of bioinformatics research have been applied to integrative machine learning with omics approaches. Identifying biomarkers is of importance to have metabolites that are robust, indicative, and predictive.

Strengths of this systematic review include the fact that most of the included studies had a low risk of bias according to QUADAS‐2 guidelines. Strict inclusion criteria allowed for meaningful comparison, with baseline characteristics being very similar amongst the included studies. In addition, there is a fairly large sample size and multiple matrices have been investigated. Analysis was carried out on three facets, providing a thorough assessment of the link between taurine and CRC for a number of criteria.

There were still limitations. For odds ratio and fold change, there was a very high heterogeneity for these outcomes. This could stem from the different matrices and the effect of methodological differences. While the sample size is sufficient, it was not very large. Ideally the sample size of each individual study would contain at least 500 samples to increase statistical power. Due to this, results may be skewed by outliers. Moreover, most of the samples were taken from patients in the Western world. Therefore, results may not be generalisable to the subcontinent or Africa for example.

## 6 Conclusions

In conclusion, there seems to be a correlation between increased taurine levels and CRC. This has now been demonstrated in multiple different studies. Likewise, the diagnostic accuracy is not diminished depending on the stage of cancer [54]. Such is the accuracy that measuring this metabolite can also differentiate benign adenomas from cancer as well. Bile acid metabolism mediated by taurine and hypotaurine shows the link between the microbiome and CRC.

Future research should focus on designing large multi‐centre randomised controlled trials looking at the effects of taurine and other products of bile acid metabolism. Carrying out a subgroup analysis may be of importance to establish which matrices are likely to be most accurate for certain metabolites when it comes to diagnosing CRC non‐invasively. Previous untargeted metabolomics studies should be refined to look at a narrower range of compounds. Analysis of taurine and other amino sulfonic acids can illustrate if there is a specific part of the bile acid metabolism pathway that is implicated in CRC samples. This will aid further understanding of the exact role of taurine. Particularly whether this amino sulfonic acid directly promotes CRC, or if it is merely increased due to the increased inflammation and oxidative stress present in the tumour microenvironment.

## Author Contributions

**Akshat Sinha:** data curation (lead), formal analysis (lead), methodology (lead), software (lead), visualization (lead), writing – original draft (equal), writing – review and editing (equal). **Liam Griffith:** data curation (equal), methodology (supporting), resources (supporting), visualization (supporting), writing – original draft (equal), writing – review and editing (equal). **Animesh Acharjee:** conceptualization (lead), funding acquisition (lead), investigation (equal), project administration (lead), resources (equal), supervision (lead), writing – original draft (equal), writing – review and editing (equal).

## Conflicts of Interest

The authors declare no conflicts of interest.

## Data Availability

All data sets are publicly available.
